# Correction: Human T-cell lymphotropic virus type 1 transmission dynamics in rural villages in the Democratic Republic of the Congo with high nonhuman primate exposure

**DOI:** 10.1371/journal.pntd.0011046

**Published:** 2023-01-06

**Authors:** Megan Halbrook, Adva Gadoth, Anupama Shankar, HaoQiang Zheng, Ellsworth M. Campbell, Nicole A. Hoff, Jean-Jacques Muyembe, Emile Okitolonda Wemakoy, Anne W. Rimoin, William M. Switzer

Fig 3 was cropped incorrectly. The full figure can be viewed here.

**Fig 3:**
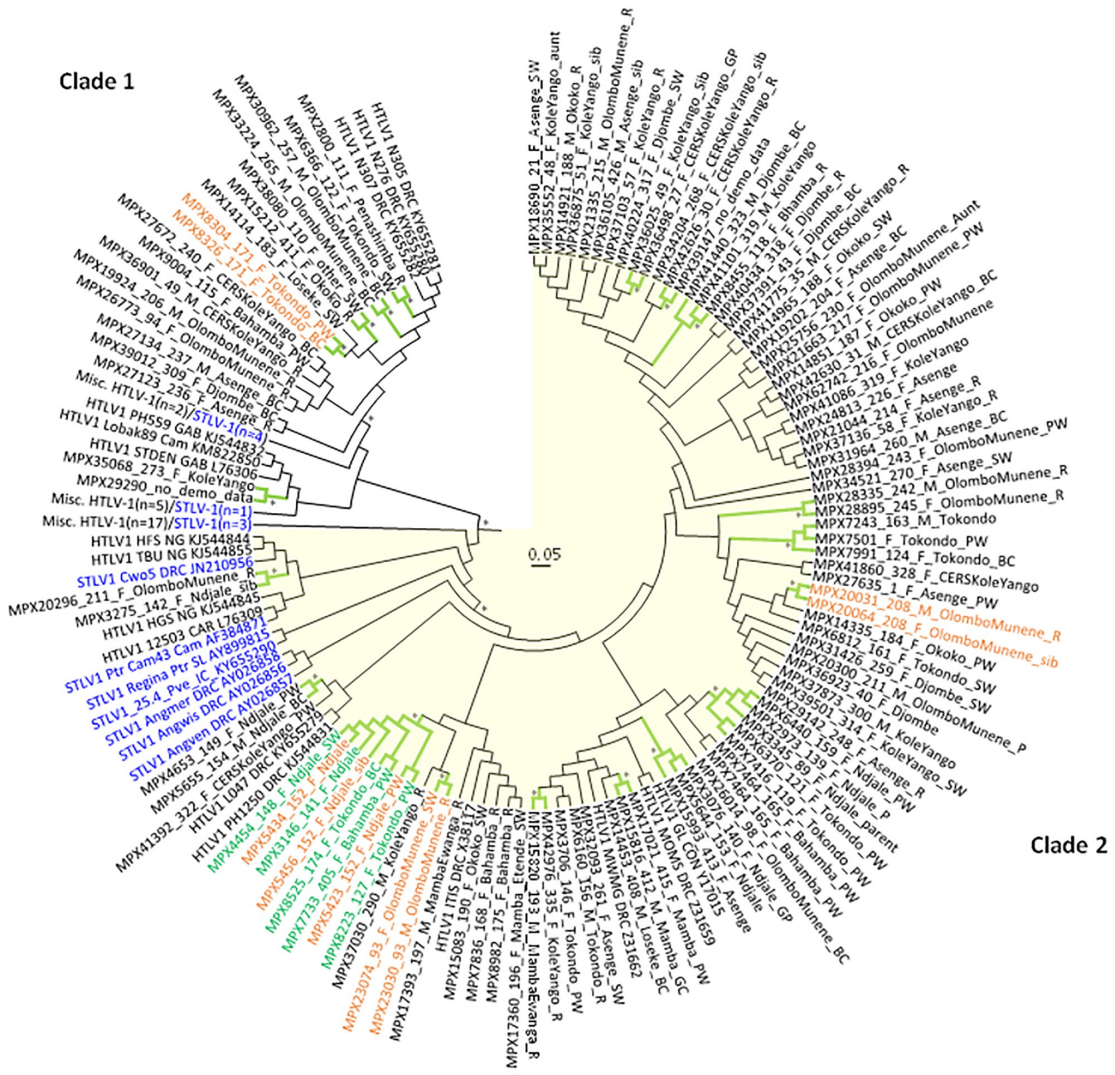
Circular Bayesian phylogenetic subtree from Fig 2 containing DRC LTR sequences. The final alignment consisted of 273 taxa with a length of 645-nt with gaps. Posterior probabilities ≥ 0.8 are indicated by an asterisk. Trees were displayed using FigTree v1.4.0. Clade 1 contains 37 taxa and is shown with a white background while clade 2 contains 127 taxa, including 85 HTLV-1 LTR sequences from our study, and is highlighted with a yellow background. The branches for those clusters with high support are colored green and marked with an asterisk. Taxa for clusters of persons belonging to the same household are colored orange and are annotated with demographics in the format: StudyID_HouseholdID_sex_village_relationship to head-of-household (HH). F, female; M, male, PW, primary wife; sib, sibling; R, responsible (same as HH); SW, secondary wife; BC, biological child; P, parent; GC, grandchild; GP, grandparent. Taxa for the eight- person cluster with the 11-bp deletion are in green; however, ones from the same household are in orange. STLV-1 taxa are blue and simian species origin are provided as three letter codes (ANG, *Allenopithecus nigroviridis*, Ggo, *Gorilla gorilla*; Ptr, *Pan troglodytes*; Pve, *P*. *vellerosus*; Cne, *Cercopithecus neglectus*; Cwo, *C*. *mona wolfii*; Cas, *C*. *ascaniu*s. Country of origin is provided in taxon name when known; Zaire is now DRC; CAM, Cameroon; IC, Ivory Coast; EG, Equatorial Guinea; CAR, Central African Republic; GAB, Gabon; NG, Nigeria; GAM, Gambia. Some branches were collapsed to improve visualization of the DRC genetic relationships. Accession numbers are provided for taxa sequences obtained at GenBank for the analysis.
