# Supplementary material for: Human T-cell lymphotropic virus type 1 transmission dynamics in rural villages in the Democratic Republic of the Congo with high nonhuman primate exposure
Source: PLoS Negl Trop Dis. 2021 Jan 28;15(1):e0008923. doi: 10.1371/journal.pntd.0008923 (PMC7872225; doi:10.1371/journal.pntd.0008923)
Supplement: S1 Table — (DOCX) [file pntd.0008923.s001.docx]

**Supporting information**

**Table S1. Animals in the Democratic Republic of Congo (DRC) included in the study participant questionnaire^1^**

| **Common name** | | | **Scientific name** | | **Common name** | | | **Scientific name** | |
| --- | --- | --- | --- | --- | --- | --- | --- | --- | --- |
| African dormouse | | | *Graphiurus* species | | Porcupine | | | *Hystrix cristata* | |
| Bat | | | *Chiroptera* order | | Rat | | | *Rattus* species | |
| Duiker | | | *Cephalophinae* species | | Reptile | | |  | |
| Elephant | | | *Loxodonta* species | | Squirrel | | | *Xerus* species | |
| Elephant shrew | | | *Macroscelididae* species | | Wild bird | | |  | |
| Gambian rat | | | *Cricetomys gambianus* | | Wild boar | | | *Sus scrofa* | |
| Mouse | | | *Mus* species | | Wild cat | | | *Felis* species | |
| Pangolin | | | *Manis* species | | Other animal | | |  | |
| **Nonhuman primates** | | | | | | | | | |
| **Common name** | **Scientific name** | | **STLV-1** | | **STLV-2** | **STLV-3** | | **STLV-4** | |
| Golden-bellied mangabey | *Cercocebus chrysogaster* | | None reported^2^ | | None reported | None reported | | None reported | |
| Red-tailed monkey | *Cercopithecus ascanius* | | 17/238 (7.1%) | | 0/238 (0%) | 0/238 (0%) | | 0/238 (0%) | |
| De Brazza's monkey | *Cercopithecus neglectus* | | 4/22 (18.1%) | | 0/22 (0%) | 0/22 (0%) | | 0/22 (0%) | |
| Greater spot-nosed monkey | *Cercopithecus nictitans* | | None reported | | None reported | None reported | | None reported | |
| Wolf's mona monkey | *Cercopithecus wolfii* | | 6/69 (8.7%) | | 0/69 (0%) | 0/69 (0%) | | 0/69 (0%) | |
| Angolan black-and-white colobus | *Colobus angolensis* | | 0/25 (0%) | | 0/25 (0%) | 2/25 (8.0%) | | 0/25 (0%) | |
| Black mangabey | *Lophocebus aterrimus* | | 0/34 (0%) | | 0/34 (0%) | 4/34 (12.1%) | | 0/34 (0%) | |
| Pygmy chimp (bonobo) | *Pan paniscus* | | 0/312 (0%) | | 6/312 (1.9%) | 2/312 (0.6%) | | 0/312 (0%) | |
| Thollon's red colobus | *Procolobus tholloni* | | 17/96 (17.7%) | | 0/96 (0%) | 1/96 (1.3%) | | 0/96 (0%) | |
| Bosman's potto | *Perodicticus potto* | | 0/1 (0%) | | 0/1 (0%) | 0/1 (0%) | | 0/1 (0%) | |
| Galago | *Galagidae* | | 0/1 (0%) | | 0/1 (0%) | 0/1 (0%) | | 0/1 (0%) | |
| Monkey, not identified | *Cercopithecidae* | | 0/1 (0%) | | 0/1 (0%) | 0/1 (0%) | | 0/1 (0%) | |
| Allen’s swamp monkey | *Allenopithecus nigroviridis* | | 21/58 (36.2%) | | 0/58 (0%) | 0/58 (0%) | | 0/58 (0%) | |
| Angolan colobus | *Colobus angolensis* | | 0/25 (0%) | | 0/25 (0%) | 2/25 (8.0%) | | 0/25 (0%) | |
| Hamlyn’s monkey | *Cercopithecus hamlyni* | | 0/5 (0%) | | 0/5 (0%) | 0/5 (0%) | | 0/5 (0%) | |
| l’Hoest’s monkey | *Cercopithecus l’hoesti* | | 0/36 (0%) | | 0/36 (0%) | 0/36 (0%) | | 0/36 (0%) | |
| Blue monkey | *Cercopithecus mitis* | | 8/45 (17.8%) | | 0/45 (0%) | 0/45 (0%) | | 0/45 (0%) | |
| Crested mona monkey | *Cercopithecus pogonias* | | 0/3 (0%) | | 0/3 (0%) | 0/3 (0%) | | 0/3 (0%) | |
| Agile mangabey | *Cercocebus agilis* | | 1/3 (33.3%) | | 0/3 (0%) | 0/3 (0%) | | 0/3 (0%) | |
| Mantled quereza colobus | *Colobus guereza* | | 0/5 (0%) | | 0/5 (0%) | 0/5 (0%) | | 0/5 (0%) | |
|  | **Total** | | 74/979 (7.6 %) | | 6/979 (0.6% | 11/979 (1.1%) | | 0/979 (0%) | |

1. STLV results were combined from five different studies in the supplemental references. PCR testing in all but the study by Meertens *et al.* was done using generic *tax* primers that detect all four STLV genotypes using dried blood spots or tissues for all monkeys and fecal samples for the bonobos followed by type-specific LTR PCR. Genotypes were determined by phylogenetic analysis of LTR sequences but varied in length. Ratio indicates number positive for each STLV/total number on animals tested followed by percentages in parentheses. Species in grey boxes were not included in the current study questionnaire.

2. None reported, testing of this species in DRC has not been reported in the literature

**References**

1. Ahuka-Mundeke S, Ahidjo A, Placide M-K, Caroline F, Mukulumanya M, Simon-Pierre N-K, et al. High prevalences and a wide genetic diversity of simian retroviruses in non-human primate bushmeat in rural areas of the Democratic Republic of Congo. EcoHealth. 2017;14(1):100-14.

2. Ahuka-Mundeke S, Mbala-Kingebeni P, Liegeois F, Ayouba A, Lunguya-Metila O, Demba D, et al. Identification and molecular characterization of new simian T cell lymphotropic viruses in nonhuman primates bushmeat from the Democratic Republic of Congo. AIDS Res Hum Retroviruses. 2012;28(6):628-35. Epub 2011/08/11. doi: 10.1089/AID.2011.0211. PubMed PMID: 21827287; PubMed Central PMCID: PMC3358107.

3. Ahuka-Mundeke S, Lunguya-Metila O, Mbenzo-Abokome V, Butel C, Inogwabini B-I, Omasombo V, et al. Genetic diversity of STLV-2 and interspecies transmission of STLV-3 in wild-living bonobos. Virus evolution. 2016;2(1).

4. Meertens L, Rigoulet J, Mauclere P, Van Beveren M, Chen GM, Diop O, et al. Molecular and phylogenetic analyses of 16 novel simian T cell leukemia virus type 1 from Africa: close relationship of STLV-1 from Allenopithecus nigroviridis to HTLV-1 subtype B strains. Virology. 2001;287(2):275-85. PubMed PMID: 11531406.

5. Mossoun A, Calvignac-Spencer S, Anoh AE, Pauly MS, Driscoll DA, Michel AO, et al. Bushmeat Hunting and Zoonotic Transmission of Simian T-Lymphotropic Virus 1 in Tropical West and Central Africa. J Virol. 2017;91(10). Epub 2017/03/17. doi: 10.1128/jvi.02479-16. PubMed PMID: 28298599; PubMed Central PMCID: PMCPMC5411610.
