# Supplementary material for: Human T-cell lymphotropic virus type 1 transmission dynamics in rural villages in the Democratic Republic of the Congo with high nonhuman primate exposure
Source: PLoS Negl Trop Dis. 2021 Jan 28;15(1):e0008923. doi: 10.1371/journal.pntd.0008923 (PMC7872225; doi:10.1371/journal.pntd.0008923)
Supplement: Attachment — Submitted filename: HTLV DRC_Rev01_2020_Response.docx [file pntd.0008923.s003.docx]

PNTD-D-20-01273 - Human T-cell Lymphotropic Virus Type 1 Transmission Dynamics in Rural Villages in the Democratic Republic of the Congo with High Nonhuman Primate Exposure

**Response to Reviewer’s questions and comments:**

**Key Review Criteria Required for Acceptance?**

As you describe the new analyses required for acceptance, please consider the following:

**Methods**

-Are the objectives of the study clearly articulated with a clear testable hypothesis stated?

-Is the study design appropriate to address the stated objectives?

-Is the population clearly described and appropriate for the hypothesis being tested?

-Is the sample size sufficient to ensure adequate power to address the hypothesis being tested?

-Were correct statistical analysis used to support conclusions?

-Are there concerns about ethical or regulatory requirements being met?

**Reviewer #1:** see Summary and General Comments

**Reviewer #2:** The authors clearly stated the hypotheses and objectives to be achieved in this study. To this end, they have described the study population, whose size in numbers ensures that the conclusions drawn are sufficiently powerful. Meanwhile, The authors should clearly specify what was the original purpose of this population-based study and why they chose this particular region of the DRC and specifically these two villages? Are there any previously published results (with the exception of those concerning SFVs) from this population-based study?

Regulatory requirements appear to have been met and clarification regarding the procedure for anonymizing the samples was sought from the authors.

Thank you for bringing this up. We have now added the original intent and purpose of this population-based study, which was to conduct zoonotic disease surveillance in two monkeypox-endemic health zones of Sankuru province, DRC. In the current manuscript, we focus on our assessment of HTLV in this study population, but we add previous publications which arose from this study design and provide further detail regarding the study site selection process.

--------------------

**Results**

-Does the analysis presented match the analysis plan?

-Are the results clearly and completely presented?

-Are the figures (Tables, Images) of sufficient quality for clarity?

**Reviewer #1:** see Summary and General Comments

**Reviewer #2:** It is important to describe the study population in more detail (mean and median age, overall range and for each gender).

Thank you for your comment. We agree and have added further classification of age in our study population both in the demographics table (now Table 2, previously Table 1) and in the results section.

The presentation of the results should be modified to make it more understandable and comparable to studies that are conducted using a comparable methodology: detection of specific antibodies by ELISA and confirmation by WB and PCR. Therefore some tables will have to be modified to be more clearly presented.

We appreciate your suggestion and, in conjunction with other suggested edits from this review, have made significant updates to all our tables to make the presentation of our results more understandable and comparable to similar studies (see new Tables 1, 2, 3). We have also updated the display of the phylogenetic subtree so the two DRC clades are more easily seen.

--------------------

**Conclusions**

-Are the conclusions supported by the data presented?

-Are the limitations of analysis clearly described?

-Do the authors discuss how these data can be helpful to advance our understanding of the topic under study?

-Is public health relevance addressed?

**Reviewer #1:** see Summary and General Comments

**Reviewer #2:** The conclusions drawn meet in part the objectives that have been defined. Indeed, some of the results could be analyzed and presented differently in a more rigorous and in-depth manner.

Thanks. We have modified the manuscript significantly as suggested by the reviewers.

--------------------

**Editorial and Data Presentation Modifications?**

Use this section for editorial suggestions as well as relatively minor modifications of existing data that would enhance clarity. If the only modifications needed are minor and/or editorial, you may wish to recommend “Minor Revision” or “Accept”.

**Reviewer #1:** see Summary and General Comments

**Reviewer #2:** We recommend that the authors make significant modifications (see Summary and General Comments section).

Thanks. We have modified the manuscript significantly as suggested by the reviewers.

--------------------

**Summary and General Comments**

Use this section to provide overall comments, discuss strengths/weaknesses of the study, novelty, significance, general execution and scholarship. You may also include additional comments for the author, including concerns about dual publication, research ethics, or publication ethics. If requesting major revision, please articulate the new experiments that are needed.

**Reviewer #1:** The authors present a very interesting work on HTLV spread at a regional level in a rural area in the DRC. They combine several types of epidemiological data and methods to provide insight into the spread of HTLV-1 within and across distant villages.

Most of my concerns relate to the phylogenetic analyses and their interpretation, which to my opinion merit some additional checks and better framing in the discussion section.

Thanks. We have modified the manuscript significantly as suggested by the reviewers.

**Abstract**

- "Questionnaire data was analyzed with serological and molecular data."

=> Questionnaire data was analyzed in conjunction with serological and molecular data.

Thanks, we changed the text accordingly.

- "with most sequences sharing an ancestral history with STLV-1 "

=> not correct formulation

Thanks, we are not sure what the reviewer means but we re-wrote the sentence to “HTLV-1 LTR sequences from 109 persons clustered strongly with HTLV-1 and STLV-1 subtype B from humans and simians from DRC, with most sequences more closely related to STLV-1 from *Allenopithecus nigroviridis* (Allen’s swamp monkey).”.

**Author summary**

lines 99-100: "Divergent STLV-1, STLV-2, and STLV-3 have been reported in monkeys and apes in DRC, increasing the likelihood of exposure to these viruses [30-33]. "

=> this sentence is not correct: the presence by itself does not increase the likelihood of exposure. Please adjust.

Thanks, this sentence in the introduction followed a sentence describing exposures to NHPs, hence it should follow logically that the existence of exposures and STLV increases the likelihood of exposure. Nonetheless, we have modified it for improved clarity to read “Divergent STLV-1, STLV-2, and STLV-3 have been reported in monkeys and apes in DRC, combined with the hunting and eating of NHPs in DRC, increases the likelihood of exposure to these viruses [30-33].”.

**Methods**

- line 128: I am surprised to see such a high level of geographical clustering of the villages, which to me seems unexpected under a random sampling scheme. Can the authors comment on this please - was the selection truly random?

Indeed, the site selection was randomized. The reviewer’s astute observation regarding the clustering of villages is a result of the fact that most human settlements in remote regions of the DRC, including this portion of Sankuru, are clustered around sparse roadways and other infrastructure vital to transport and communication, which could facilitate transmission of disease.

- can the authors comment on the quality of the samples? I am not a wet-lab expert, but I can imagine that it is not easy at all to preserve samples in high quality conditions in remote areas, in that climate, and over such long time frames. Was a quality check on the sample integrity performed? Could sample degradation underlie for example that the reported estimates represent lower end estimates of the true HTLV prevalence? If so, this should be discussed.

We appreciate your concern. After specimen collection and processing the plasma and buffy coats were stored locally in ultralow freezers prior to shipping to the US as already described in the methods. Given that we were able to identify HTLV and SFV antibodies in the samples at prevalences comparable to other studies in Africa these result show that the quality of the samples did not appear to have been compromised. DNA quality was determined using spectrophotometry and by PCR detection of B-actin gene sequences in specimens used for *tax* and LTR sequence generation. B-actin was detected in all DNA samples. We now provide this information in the paper in the methods (lines 178-179, 188-189) and results (lines 302-304).

- "Genetically related sequences were identified using BLAST searches (http://www.ncbi.nlm.nih.gov/BLAST/) and added to the analyses for comparison along with HTLV references"

=> the authors should be more clear as to how the dataset was compiled. How many BLAST hits were retained for each of the query sequences? Why was there an apparent need to separately add reference sequences and other HTLV sequence data from the DRC?

Identifying genetically related sequences in public databases is a standard procedure for conducting transmission and evolutionary analyses, especially when trying to determine the origin of HTLV-1 in DRC, which would include publicly available sequences from DRC and other African countries. We now briefly add this information to the methods and provide references for this strategy in other studies. We also add in the results the number of total unique sequences in the final alignment identified by the BLAST search of GenBank.

- why did the authors choose for a constant-rate BD tree prior instead of a (non-parametric) coalescent model? Also, which priors were specified on the parameters of the BD-process? this should be specified, as well as the rationale for each.

We and others (Leendertz et al. JVI 2010) have previously used the birth-death (BD) model for successfully inferring PTLV evolutionary histories. We did not use a constant rate as suggested by the reviewer but instead used a lognormal relaxed clock model as in our previous studies. We now site these previous publications and provide the xml file in the supplementary material which includes the parameters used.

- fig 2b: The branch lengths seem quite close to a multiple of 1 - this suggests that there was very little diversity in the alignment, and that the temporal aspect may be unreliable. The authors should evaluate whether or not there is significant temporal signal in the data to calibrate the clock model. This can be done as in Trovao et al Virus Evolution, 2015, 1(1): vev016) by comparing the rate estimate with the expectation in the absence of temporal structure. Below the tweaks that need to be specified in the XML:

1) make sure that each taxon has an associated leafHeight parameter [this goes in the treeModel block]:

2) add an operator that permutes the tip dates during the run, giving the evol rate expectation in the absence of temporal signal

3) to check whether all went well, log the tip-ages [and perhaps inlcude a tip age in the screen-logger element for a quick visual check]:

in the fileLog-element:

and for the screenLog:

The usual criterium is non-overlapping 95% credible intervals. The test for temporal signal should be reported, perhaps using a supplementary figure.

We did not conduct a time-calibrated phylogeny which is why there is not a time axis and no date information in the manuscript. The reviewer is correct that HTLV evolves slowly which can impact inferring divergence dates but which we did not determine in our study. We did conduct analyses to insure there was good phylogenetic signal in the alignment using multiple methods, including the composition Chi square test, likelihood mapping and the online tool Guidance as described in the methods. Furthermore, it was recently shown that checking for temporal signal as done in Trovao et al. suggested by the reviewer, when using the BD model is not recommended because the sequences, number of sequences, and sampling times are ignored (see Boskova et al. Vir Evol 2018).

- The authors should justify their choice for the relaxed clock model: I believe that the HTLV evolutionary rate is linked to its transmission rate (Salemi, PNAS). If what the authors refer to as 'potential transmission clusters' indeed represent human-to-human transmission, I would think that one can expect 2 rate-distributions with different means operating on the phylogeny: a slower rate on the internal branches that represent evolution in the reservoir (on which I expect less frequent transmission), and a higher rate in clades that represent human-to-human transmission. In such situations the relaxed clock model will perform poorly (see a.o. Worobey et al Nature 2014), and a local clock model will be better suited. While the latter require prior knowledge on which branches to expect a rate difference, a random local clock model may offer a valuable 'naive' alternative (https://pubmed.ncbi.nlm.nih.gov/20807414/). Both can be set up using BEAUTi.

Relaxed molecular clocks are typically used for inferring retroviral phylogenies (Wertheim and Worobey PLOS comp biol 2009; Worobey et al Nature 2016; Salemi et al PLOS One 2008) and which we and many others have previously used to accurately infer the evolutionary histories of PTLV (Mossoun et al. JVI 2017, Calvignac-Spencer et la. EID 2012, Leendertz et al. JVI 2010, Sintasath et al. Retrovirol 2009 etc). We now provide these PTLV references in the methods. The Worobey et al. article cited byt the reviewer describes a study of influenza which has a substitution rate much closer to HIV (~10^-3^) than HTLV (~10^-6^ – 10^-7^) and which spreads via cell-free virus vs cell-associated viruses like PTLV. The Salemi PNAS article describes a faster evolutionary rate of HTLV-2 in IDUs (10^-4^) compared to endemically infected Amerindians (10^-7^) using older methods and which to our knowledge has not been re-analyzed using Bayesian inference for confirmation nor has this been reported for HTLV-1. Furthermore, Afonso et al. Retrovirol 2019 reported not finding a difference in the evolutionary rates of HTLV/STLV.

- the substitution model should be specified

The nucleotide substitution model was already provided in the methods on page 10, lines 212-214.

- line 209: "significant ESSs" => reword to "high ESSs" (there is no criterium to bin ESS as significant or not to my knowledge)

Thanks, we removed “significant” from this sentence.

- fig2a looks like an unrooted phylogeny, but it is not mentioned with which models/software this was inferred?

Yes, this is an unrooted phylogeny. We have added to the methods and figure legend that trees were displayed with FigTree v1.4.0 and provide the URL for this software. We already provide in the figure legend that the trees were inferred using Bayesian inference the details of which are already provided in the methods.

- fig2b: this is not a nice presentation of the phylogeny. Better in this case is to use a rectangular layout. Also, the long tip labels hinder interpreting the tree - if these can be shortened to only contain the necessary information. The authors should use a time axis; why else estimate a time-calibrated phylogeny? The 2 major clades that are discussed should be clearly indicated. The purple for highlighting the clades with good support should be more bright to more clearly distinguis these clades from the rest. I miss a look at how long these cluster's MRCA goes back in time? Are they recent, old, a mixture of both? To when does the the 89-HTLV-clade MRCA date?

We respectfully disagree with the reviewer. We tried a horizontal tree layout for the subtree in figure 2B but there are so many taxa that the text becomes too small to clearly read. A circular tree as used in figure 2B is more commonly used in this situation as we and others have done in other publications (for examples see Switzer et al. Retrovirology 2012, Ahuka-Mundeke et al EcoHealth 2017). We do not think the taxa labels interfere with interpretation of the phylogeny but rather are required for better correlation and understanding of the written results when referring to the tree. We did not conduct a time-calibrated phylogeny which is why there is not a time axis and no date information in the manuscript.

Thanks for your other suggestions. We now indicate the two large clades in Fig 2B and change the color of the purple branches to bright green, so they are more easily visible as suggested.

- about the potential transmission clusters: there clearly is intermixing of STLV with HTLV, suggesting rather frequent cross-species jumps. But is it no also true that the extant diversity of the STLV reservoir is severely underrepresented (undersampling of the STLV reservoir)? If so, what look like transmission clusters can very well be separate cross-species transmission events for which the source is simply not sampled. What arguments do the authors have against this scenario? They mention that 3 ang cluster as a sister clade to a 89-HTLV clade, but I don't see how this backs the idea that this 89-HTLV clade represents human-to-human transmission.

A random local clock model can add evidence for either scenario: if there is a rate shift on the branch leading to the MRCA of the 89-HTLV clade such that this evolves more at a higher rate then the branches pertaining to reservoir evolution (of which I suppose less frequent transmission), this would be evidence against multiple cross-species transmissions.

Yes, under sampling of STLV-1 is a limitation of the study, which we already describe in the discussion on pages 23-24. No other STLV-1 from DRC or Africa clusters within or ancestral to the large 89-member clade suggesting that transmission may have occurred from STLV-1ang in the past followed by human-to-human transmission. Absent other STLV-1 sequences from additional NHPs in DRC this is the most parsimonius explanation of the results. This is analogous to the cosmopolitan HTLV-1a genotype for which a parental STLV-1 sequence has not yet been identified. HTLV-1a must have originated from a closely related STLV-1 after introduction into humans and then spread within humans. We now add this point to the discussion. Repeating the phylogenetic analysis with a random molecular clock as suggested by the reviewer may help determine evolutionary rates along the branch but is not the focus of the study and would not resolve the observed phylogenetic relationships or address transmission. Furthermore, Afonso et al. Retrovirol 2019 reported not finding a difference in the evolutionary rates of HTLV and STLV.

- the NHPs in the questionnaire: many have not been shown to possibly harbour STLV-1 (supp table). I think that this substantially weakens the argument in favor of community spread based on "the lack of an association of NHP exposures and HTLV WB positivity" as NHP exposure via this measure does not very directly suggests exposure to STLV-1.

Also, Can the lack of an association between NHP exposure and HTLV WB positivity be explained by higher sensitivty of the serological tests for HTLV-1 compared to HTLV2/3 (which are found in 4 of the NHP species)? This should be better discussed.

Thanks for your comment. We have updated Table S1 with additional details of NHP species from DRC that have been tested from additional studies using PCR. Eleven monkeys from DRC have not been identified with STLV-1 infection and eight were not included in our study questionnaire. The absence of STLV-1 likely reflects the complete absence in those species or the testing of small numbers of a species like some *Cercopithecus* species and the potto and galago prosimian monkeys. STLV-2 has only been found in bonobos in Africa and STLV-3 is typically more prevalent than STLV-2 but less prevalent than STLV-1. STLV-3 was found in 5 NHP species, four of which were included in our questionnaire. Our conclusion of community spread is based on the phylogenetic results not on the presence of STLV-1 in monkeys in DRC. Of the 165 HTLV-1 WB-positive samples, 110 (66.7%) were PCR-positive and 55 were PCR-negative (33.3%). All 110 PCR-positive samples had HTLV-1 sequences - not STLV-1 sequences – of which 50 (45.5%) were in potential transmission clusters strongly suggestive of community spread from person-to-person.

Serology is used to better screen for divergent viruses such as STLV/HTLV in large volumes of specimens since cross-reactivity, including indeterminate results, is better to detect these and unknown variants. The commercial assays we used include antigens to both HTLV-1 and HTLV-2 and we now add that information to the methods since it may not have been clear. Serology is also better than PCR when sequences from the variants are not available for their detection, hence our use of generic *tax* primers for detecting divergent PTLV in all the seroreactive specimens, including the indeterminates. We already describe in the methods that this strategy of broad screening using serology and generic PTLV PCR has been used successfully by many groups for the identification of novel HTLV-3 and HTLV-4 genotypes and is more cost effective that performing only PCR on thousands of samples, which has its own limitations. Nonetheless, commercial serologic assays have limited evaluation for detecting STLV-2, -3 and -4. We now add in the discussion this potential limitation of using commercial assays for HTLV-1 and HTLV-2 antigens to detect STLV-2, -3, and -4.

- what is the cluster definition? Only based on support? Why not also take some measure of time/genetic distance into account?

Thanks for your suggestion We initially defined a cluster based on posterior probabilities of the nodes ≥ 0.8 but now add *≥* 99% shared percent nucleotide identity. This resulted in exclusion of one pair and one triad from the original analysis that did not meet this definition. We have added this information to the methods in lines 228-230 and adjusted the results accordingly.

**Results**

- line 236: which levels? Pleeas specify in the Methods section, or detail here.

This is an introductory sentence. The specific results follow in the next sentence.

- line 243: "Only a marginally significant association

244 between SFV and HTLV co-infection was observed (χ2 p-value= 0.0534).".

=> Is a Fisher exact test not better suited here? Thanks. We now use the Fisher’s exact test with the relevant data.

> m <- matrix(data = c(3,14,162,2872), nrow = 2, ncol = 2, byrow = T)

> m

[,1] [,2]

[1,] 3 14

[2,] 162 2872

> fisher.test(x = m)

Fisher's Exact Test for Count Data

data: m

p-value = 0.06041

alternative hypothesis: true odds ratio is not equal to 1

95 percent confidence interval:

0.6925923 13.7944348

sample estimates:

odds ratio

3.795586

**Discussion**

- "The overall HTLV seroprevalence in our population was 5.4% .... In accordance with our study’s 4.2% HTLV-1 prevalence"

=> contradiction?

Apologies for any confusion here. The 5.4% prevalence is for seroreactivity to any HTLV (1, 2, and those positive but untypeable), whereas 4.2% denotes the prevalence of HTLV-1 specifically. We have updated the text to clarify this distinction.

- " Nonetheless, we also did not find any evidence of recent STLV-1 infection in our population despite high NHP exposure and previous identification of SFV infection in this same group."

=> I perhaps overlook it, but how was the recency of HTLV infection established?

We apologize for any confusion. We did not measure recency as is done for HIV and other pathogens using serologic assays. We meant that there is not a direct link in the phylogeny of an HTLV-1 sequence from DRC to an STLV-1 sequence as would be expected with a recent zoonotic infection. Rather STLV-1s were more distantly related, including STLV-1ang, the sister clade to the large DRC HTLV-1 clade. We now provide a new sentence in the discussion in lines 549-551 to further clarify this point.

**Supplementary info**

what does '0/1' under STLV's reported refer to? Please add this information.

This means that only one animal was reportedly tested and that is was negative for STLV. We have modified the table considerably to include ratios and percentages of STLV results for each simian species with footnotes and provide a references section.

**Reviewer #2:** In this manuscript, the authors studied the presence of HTLV infection in villages from a specific region of the Democratic Republic of the Congo (DRC).

It is important to perform such studies in DRC, a very highly populated country of central Africa where relatively few studies have been performed on such viruses in humans. This is an interesting paper that combines serological, viro-molecular and phylogenetic findings and provides some data on the transmission dynamics of HTLV-1 in remote villages in DRC with populations exposed to frequent contact with non-human primates.

This study was carried out by teams from the United States, one being headed by William M. Switzer, one of the leading scientists working on zoonoses in particular retroviruses such as HTLV and Simian Foamy Viruses.

We were therefore quite surprised by a certain lack of precision, particularly in the Methods and Results sections presented in this study.

In this context, the quality of the manuscript should be greatly improved especially by answering the following remarks and questions.

Major comments:

1) Population description.

Page 7 lines 126 and following.

-The authors should clearly specify what was the original purpose of this population-based study and why they chose this particular region of the DRC and specifically these two villages? Are there any previously published results (with the exception of those concerning SFVs) from this population-based study?

This question was duplicated in the reviewer form, so we are repeating our answer here for clarity. Thank you for bringing this up. We have now added language around the original intent and purpose of this population- based study, which was to conduct zoonotic disease surveillance in two monkeypox-endemic health zones of Sankuru province, DRC. In the current manuscript, we focus on our assessment of HTLV in this study population, but we now provide previous publications which arose from this study design and provide further detail regarding the study site selection process.

- In addition, they used only 3,051 of the 4,573 eligible individuals. Can the authors specify whether they think this fact may have introduced a bias in the study results?

We created an analytic subset of all participants for the current analysis (e.g., all participants with both blood samples and completed surveys) to allow for an examination of epidemiologic and phylogenetic markers of HTLV in the study population. In order to examine the potential for bias introduction from use of this subset, we have shown in Table 2 a comparison of all study enrollees—i.e., those with and without eligible specimens—according to demographics of interest. We have included percentages to show the reader where differences appear between the full and analytic samples. We have also now included a chi-square test of proportions to show statistically that the three demographic variables appear to vary between these groups (age, ethnicity, and health zone). We have also now included a discussion of this potential selection bias in the discussion section. Thank you very much for the suggestion.

- What were the types of populations living in these areas (tribes, ethnic groups, ..)?

Our analytic sample was comprised primarily of two ethnic groups, Batetela (30%) and Ohindo (55%), which mirrored the total study population. The ethnic composition of our study population can now be found in Table	2.

- Page 7, Line 136: Could the authors describe in more detail the anonymization procedure used for the samples they collected?

We have added a sentence describing our use of unique and randomly assigned ID numbers to anonymize participant data.

- How much blood did they sample from children, especially the younger ones, and what method did they use (EDTA tubes or DBS)?

As noted in the biological specimen collection portion of the methods section, all blood was collected using EDTA tubes. For enrolled children under 10kg of weight, a maximum of 3 mL of blood was collected.

2) Results.

- It is important to describe the study population in more detail (mean and median age, overall range and for each gender).

Thank you for your comment. We agree and have added further information on the age details in our study population both in the demographics table (now Table 2, previously Table 1) and in the results section.

- It is not clear for the reviewer why the authors grouped the results after the WBs, considering the general HTLV item for sero-epidemiological analysis. Indeed, such HTLV includes, according to Table 1; HTLV-1, HTLV-2 and non-typeable HTLV. First, the authors should eliminate for their analysis those individuals diagnosed with HTLV-2 WB serology. Second, the results for non-typeable WB should be detailed: how many cases were HTLV-1/2 with reactivities to both MTA-1 and K55? It is very likely that in such a large series, some WB will exhibit such a pattern. Third, what does “like” mean after HTLV-1 and HTLV-2? According to the manufacturer of the WB kit, this is not mentioned. Finally, how many HGIP were observed among the 85 indeterminate pattern?.

Thanks for your suggestions. We grouped the WB results similarly in other publications by our group. For example, see PTLV emergence PNAS 2005 Wolfe et al and Dual SFV-HIV-1 infections in persons from Cote d'Ivoire Switzer et al PLOS ONE 2016. We respectfully disagree with the reviewer and prefer to include the full spectrum of HTLV WB results in the manuscript as this defines the potential HTLV infections in our population and provides new data on HTLV infection in DRC and Africa. The Wolfe et al. paper cited in the methods (#7) describes the WB classifications. We have now added them to the current manuscript on lines 166 – 170, which includes “HTLV-like”. Since we and others have identified STLV infection in humans using these serological assays that give HTLV-1 and/or HTLV-2 WB profiles we prefer to describe the WB results using “like” until sequence analyses are conducted to genotype the infection. We now add a brief description of the untypeable WB results to the results, which included 20 samples with reactivity to MTA-1 and K55. We also now add the number of HGIPs (n=6) to the results.

- The authors should first focus their work on the HTLV-1 virus, which is by far the most prevalent and important virus in their population.

Thanks. We are not sure what the reviewer is requesting. Since most infections were HTLV-1 in an area where retroviral zoonoses have been documented the focus of the results and paper is on HTLV-1 and their origin and transmission in our study population. We hope our revision has addressed this suggestion.

- Another question is therefore to know what are the criteria to consider that a person HTLV-1 infected with HTLV-1 in their study: either the authors use a serological criterion only with HTLV-1 in WB and HTLV-1/2 including those for which the MTA- band 1 is stronger than the K55 band, or they also add a PCR criterion by including non-typeable profiles for which the HTLV-1 PCR (s) are positive. Authors should select and clearly define their positivity criteria.

Thanks. We have already defined in the methods in the statistical analyses how infection status is defined. We chose to use WB-positivity (HTLV-1-like, HTLV-2-like, HTLV untypeable) as infected persons and indeterminates as not infected. We now add in the methods on lines 172-173 that “Persons with HTLV WB-positive results were considered infected regardless of PCR status.” We did not score the intensity of the K55 and MTA-1 bands to conduct the analysis suggested. Twelve of the untypeable WB samples were PCR-positive, including 8 with reactivity to both gp46 proteins and four without. We now add these HTLV-untypeable WB and PCR results to lines 302-303 in the results section.

Thus, the abstract, methods, results and Table 1 should be greatly modified based solely on analyses of HTLV-1 results.

No other changes in the manuscript are required since our definition of infection is unchanged.

This would provide more robust and comparable data with other studies carried out elsewhere in the world and particularly in Africa.

We have used the same WB criteria for other HTLV studies in Africa (please see Dual SFV-HIV-1 infections in persons from Cote d'Ivoire Switzer et al PLOS ONE 2016; PTLV emergence PNAS 2005 Wolfe et al.). We feel the use of HTLV-positive but untypeable permits better classification of the HTLV seroreactivity, especially in studies examining retroviral emergence and cross-species infections. It is definitely better than calling them indeterminates for which most studies have shown these to be PCR-negative in contrast to our study that shows the majority of the WB-untypeables were PCR-positive. Further, these results are provided separately in our paper and could easily be extracted for comparison in other studies.

Subsequently, the authors may also describe data from the few individuals with HTLV-2 profile. It would be ideal to have specific HTLV-2 PCR as welle as others that can detect HTLV-3 and 4, as the authors know that people infected with the latter two retroviruses may have WB serologies, sometimes resembling HTLV-2. Can the authors comment on this, provide new data and modify their results?

Thanks for this suggestion. We have now added the WB profiles for the three HTLV-2-like WB samples in the results on line 255-257. None of these samples were PCR-positive as we describe in the results and discussion already. We now add to the discussion in lines 551 the potential limitation that the available commercial assays may not accurately detect infection with all strains of HTLV-3 and -4 as they have limited validation to do that and that new HTLV tests that include HTLV-3 and HTLV-4 antigens are needed for their detection.

Page 31, Table 1: In addition of the number of HTLV-1 positive samples, the authors should add the resulting prevalence of HTLV-1 in each category.

Thanks for your suggestion. We have added the percentage for each category as requested.

Page 32, Table 2: This table presents the odds ratio of being HTLV-1 in a household with at least one HTLV-1 infected member. Given that the prevalence of HTLV-1 increases with age and is higher among women, the results showed in this table are of limited interest and can lead to biased interpretation. Indeed, caution should be taken as the reference group here is the biological child. The odds ratio of being infected for the primary and secondary wife will obviously depend on which one is the mother of the child.

We agree with the reviewer and struggled with the same issues the reviewer mentions here. Due to the inherent links between traditional familial structures/household roles in Sankuru with age and sex, the significant correlation of these three variables prevented us from properly teasing apart the impact that household roles and family/social structure may have had on HTLV serostatus as a pathway for potential person-to-person transmission. As such, we have clarified our discussion of this limitation and decided to remove this table from the manuscript, focusing this piece of the study instead on the phylogenetic cluster analysis.

Page 32, Table 3: As demonstrated by the authors, the exposure to nonhuman primates and the type of exposure are significantly associated with gender. Additionally, there is a significant difference of HTLV-1 seroprevalence between sex. The analysis of NHP exposure as potential risk factors of HTLV-1 transmission should therefore be at least adjusted on sex, but can also be adjusted on both age and sex. Table 3 should therefore give sex-adjusted OR and the authors should add the number of HTLV-1 positive samples in each category.

We thank the reviewer for their comments and have updated this table accordingly with an age- and sex- adjusted OR provided for each exposure type, as well as the number of HTLV positive samples in each category.

**Minor comments:**

**Introduction**

Page 4, Line 67: The authors can also quote here a recent paper published on the molecular epidemiology of HTLV-1 especially in Africa (Afonso et al., Retrovirology 2019).

Thanks for your suggestion. We have added this reference as requested.

**Methods section**

Study population sub-section

Questionnaire administration sub-section

Page 7, line 141: Regarding the data collected on exposure to non-human primates, how did the authors assess the bias related to unauthorized hunting activities that could result in the minimization of these activities?

To reduce social desirability bias related to answering questions on animal hunting (specifically unauthorized hunting activities of specific species), all surveys were administered by local, trained interviewers and were anonymized as described in the updated methods section. Furthermore, questions regarding animal exposures were never prompted or asked in a framework of legality, nor were certain species grouped by their vulnerability or conservation status. We now add this information to the methods. We suspect that a large majority of our study population would not be aware of the presence of certain species on unauthorized hunting lists.

Serology, PCR, and phylogenetic analysis sub-section

Page 8, Line 168: Considering the age of the samples collected in 2007, did the authors verify the quality of the DNAs by amplifying a housekeeping gene before carrying out targeted amplifications of the different HTLV genes/fragments?

Thanks for your suggestion. The other reviewer raised the same issue. After specimen collection and processing the plasma and buffy coats were stored locally in ultralow freezers prior to shipping to the US as already described in the methods. Given that we were able to identify HTLV and SFV antibodies in the samples at prevalences comparable to other studies in Africa these result show that the quality of the samples did not appear to have been compromised. DNA quality was determined using spectrophotometry and by PCR detection of B-actin gene sequences in specimens used for *tax* and LTR sequence generation. B-actin was detected in all DNA samples. We now provide this information in the paper in the methods (lines 178-179, 188-189) and results (lines 302-304).

**Results**

NHP Exposer sub-section

Page 12: To our knowledge, it is mainly the bite of an PNH that has proven to be the main ”zoonotic” risk factor for being infected with HTLV-1 in these village populations. Authors should isolate this factor in their analysis and not include it with "scratching" and "playing" activities.(see Table 3).

We thank the reviewer for this comment. When we initially separated NHP biting, playing, and scratching in our analysis, we found that no HTLV-positive study was either bitten or scratched by an NHP within the last month. As such, we were unable to draw conclusions about the potential impact of these exposure types in Sankuru but have now clarified this limitation for the reader. An updated Table 3 with each of these exposure types has been included in the revision, and the text of the paper has been altered accordingly.

HTLV PCR and Phylogenetic Analyses sub-section

Page 13: Concerning the PCR results, the authors must, for each serological profile in the WB (HTLV-1, HTLV-2, HTLV-1/2, HTLV not typeable, indeterminate including the HGIP profile,...), give the results of the different PCRs (specific for HTLV-1 at least, or also for HTLV-2, 3 and 4). This would make it possible to know the % of positive PCRs in each category. A table of this kind is available in the manuscript by Djuicy et al. PloS.TND, 2018, Table 1.

Thank you for your comment. To address this, we are including a new Table 1 that delineates the results of the different PCRs for each serological profile in the WB.

Page 14: To my knowledge, the first demonstration that Allenopithecus nigroviridis are infected by HTLV-1 genotype b, has been published by Meertens et al. in Virology in 2001. This paper describing the 3 strains included in the phylogenetic analysis (Angwis, Angmer, Angven) should be quoted and discussed in this article.

You are correct. We apologize for not including this important reference. We now include the reference throughout the manuscript when these three sequences are described. We also now provide a more thorough discussion of our results and STLV-1 in *A. nigroviridis.*

The different main clades of the phylogenetic tree (2B) should be presented more clearly with separations highlighting the different clades.

Thanks for your suggestion. We now indicate the two large clades in Fig 2B with shading and names, so they are more easily visible as suggested.

Pages 14-17: The section between lines 314 and 367 is long and often difficult to read. The authors might consider simplifying and shortening it.

We appreciate the feedback and have shortened this section of the results to more clearly explain patterns of interest to the reader.

**Discussion**

Page 18: In the introduction of the discussion, the authors could write a few lines on what is currently known about HTLV-1 related diseases in DRC (ATL, HAM / TSP, ..) and the situation of donors blood or pregnant women.

We have modified the introduction of the discussion to describe more thoroughly the history of HTLV in DRC, including prevalence rates from previously published literature that examine Congolese populations specifically, and a mention of the relative lack of knowledge surrounding HTLV-1 related diseases in DRC given several limitations of the healthcare system.

In general, authors should be cautious when referring to review articles especially for Africa and HTLV-1. Indeed, very often the methodologies used, such as the tests employed and the populations studied (pregnant women, blood donors, hospitalized patients, general population) are very different and not comparable. This applies in particular to the prevalence of HTLV on the African continent (see below).

We very much appreciate the reviewer’s comment and have taken great care to revise this portion of the discussion to more accurately depict the various study populations from whom information about HTLV in Africa has been reported.

Page 18, line 396: To our knowledge, it is not reasonable to write that the prevalence rate of HTLV on the African continent averages 10%. As the authors are well aware, it is almost impossible for HTLV-1 to give an average for a large region or even a country because of very different prevalence in different geographies. Regional microepidemiology distribution is a main feature of HTLV-1 distribution; this has been well demonstrated in parts of African and southern Japan. Moreover, in the general adult population, even in remote villages, the highest prevalence very rarely reaches 5 to 15%.

As far as we know, this prevalence has only been observed in part of the rural population of Gabon. The data obtained in this study are quite comparable and constitute one of the highest prevalence of HTLV-1 observed in Africa. This finding could be clarified by the authors, at least in the abstract, results and discussion. Please comment on this topic and modify the sentence on line 396 accordingly.

Again, thank you for the thoughtful comment. We are cognizant of the large geographic and demographic variability that leads to regional microepidemiologic distribution of HTLV and have made significant updates to the discussion section to treat this variability with more care. We have also made note of the relatively high prevalence of HTLV-1 in our study population in the abstract now.
